# Long‐Term Growth Outcomes of Children With Type 1 Diabetes According to Glycemic Control and Use of Continuous Glucose Monitoring: A Retrospective Cohort Study

**DOI:** 10.1155/pedi/9111583

**Published:** 2026-03-02

**Authors:** Sujin Kim, Seo Jung Kim, Kyoung Won Cho, Kyungchul Song, Youngha Choi, Hyun Wook Chae, Junghwan Suh

**Affiliations:** ^1^ Department of Pediatrics, Severance Children’s Hospital, Yonsei University College of Medicine, Seoul, Republic of Korea, yonsei.ac.kr; ^2^ Department of Pediatrics, Gangnam Severance Hospital, Yonsei University College of Medicine, Seoul, Republic of Korea, yonsei.ac.kr

**Keywords:** children, continuous glucose monitoring, glycemic control, height standard deviation score, linear growth, type 1 diabetes mellitus

## Abstract

**Background:**

Chronic diseases such as type 1 diabetes mellitus (T1DM) may alter linear growth; however, reports regarding growth in children with T1DM have been inconsistent. This study aimed to investigate the height and growth velocity of patients with T1DM, and whether they were affected by various factors 5 years after the diagnosis.

**Methods:**

This retrospective study included patients with T1DM between October 2005 and May 2022, with a follow‐up period of at least 1 year. Patients with diabetes, thyroid disease, celiac disease, or any other chronic disease were excluded. We compared the mean height standard deviation score (H‐SDS) and growth velocity between groups divided based on glycosylated hemoglobin (HbA1c) levels and use of continuous glucose monitoring (CGM) systems.

**Results:**

Among the 150 patients, 45.3% were male, with a mean age at diagnosis of 7.8 ± 3.6 years. At diagnosis, the mean H‐SDS was 0.38 ± 1.11. In males, H‐SDS significantly decreased overtime, with an estimated slope (*β*) of −0.054 (standard error [SE] = 0.013, 95% confidence interval [CI]: −0.079 to −0.029, *p*  < 0.01). The decline in H‐SDS was more pronounced in the poorly‐controlled group (mean HbA1c ≥7.0%) compared to the well‐controlled group (mean HbA1c <7.0%; *β* = −0.081, SE = 0.016, 95% CI: −0.112 to −0.050 vs. *β* = −0.007, SE = 0.020, 95% CI: −0.047 to −0.033, *p*  < 0.01). Among males using CGM, the decrease in H‐SDS over the 5‐year follow‐up was significantly less than that observed in the non‐CGM group (*β* = −0.012, SE = 0.023, 95% CI: −0.057 to −0.034 vs. *β* = −0.072, SE = 0.015, 95% CI: −0.101 to −0.042, *p* = 0.03). In the multivariable linear mixed model analysis, younger age at diagnosis (*β* = −0.009, 95% CI: −0.017 to −0.002, *p* = 0.02), female (*β* = 0.067, 95% CI: 0.033 to 0.100, *p*  < 0.01) and lower HbA1c levels (*β* = −0.026, 95% CI: −0.038 to −0.015, *p* < 0.01) were significantly associated with greater improvement in H‐SDS over 5 years.

**Conclusion:**

Glycemic control and CGM use positively affected linear growth in children with T1DM, especially in males. CGM use was associated with improved growth outcomes, which suggests that glucose monitoring may help mitigate the adverse effects of poor glycemic control on growth.

## 1. Introduction

The incidence of type 1 diabetes mellitus (T1DM) in children has increased significantly, with reports indicating 32.39 cases per 100,000 children in 2020 compared to 19.73 per 100,000 children during the pre‐COVID‐19 era [1]. As a chronic condition in children, T1DM poses concerns regarding its impact on growth during the critical developmental years; however, studies on the height of children at the time of T1DM diagnosis have yielded inconsistent results. Some studies report that children with T1DM are taller than the general population at diagnosis or up to 3 years before the diagnosis [2–5], while other studies found no significant height difference compared to their healthy peers [6–8]. Brown et al. [9] observed that children diagnosed with diabetes mellitus between the ages of 5 and 10 years tended to be taller than their peers without diabetes.

After T1DM onset, the height standard deviation score (H‐SDS) and linear growth rate decreased consistently in cohorts of patients with T1DM [10]. Previous research has indicated that inadequate glycemic control and/or long‐standing T1DM may adversely affect linear growth in children [11], which is attributed to changes in the growth hormone/insulin‐like growth factor (GH/IGF‐1) axis. Studies have also shown that the final adult height may be significantly reduced in children with inadequate diabetes management, especially in those with glycosylated hemoglobin (HbA1c) levels >8.0% [12]. Delayed puberty and impaired pubertal growth, particularly in females with T1DM, are also associated with poor glycemic control [13, 14].

The use of continuous glucose monitoring (CGM) has become widespread and is recognized as a contributing factor in improving glycemic control and reducing HbA1c levels [15, 16]. Numerous studies have demonstrated the beneficial impact of CGM on various aspects, such as increasing the time in range of glucose levels and lowering the mean average of HbA1c by 0.2% [17]. However, while the utilization of CGM potentially contributes to improved growth because of the positive effect on glycemic control, there remains a paucity of research investigating the relationship between CGM utilization and linear growth.

The aim of this study was to analyze changes in the H‐SDS and growth velocity using the linear growth of individuals with T1DM following diagnosis, which was stratified by sex, using a single‐center cohort based on the extent of glycemic control and utilization of CGM.

## 2. Methods

### 2.1. Design and Study Population

This retrospective study included 162 patients diagnosed with T1DM who began insulin treatment between the ages of 0 and 18 years, from October 2005 to May 2022. All patients were followed for at least 1 year at the Outpatient Pediatric Endocrinology Unit of Severance Children’s Hospital, a tertiary center with two branches in South Korea. Patient identification was conducted using the Severance Clinical Research Analysis Portal (SCRAP), a web‐based medical data analysis platform that integrates order communication system (OCS) information and electronic medical record (EMR) data stored in pseudonymized form. We searched SCRAP for pediatric patients with medical records from October 1, 2005—when the EMR system was implemented at our institution—to May 2022. All the patients with diagnosis codes related to diabetes mellitus were identified, and those diagnosed with T1DM were selected for inclusion. The diagnostic criteria for T1DM were based on the guidelines of the International Society for Pediatric and Adolescent Diabetes (ISPAD) [18] and the Korean National Health Insurance criteria. Diagnosis required fulfillment of at least one of the following: presence of diabetes‐related autoantibodies (e.g., islet cell antibodies, glutamic acid decarboxylase 65 (GAD) antibodies, or insulin antibodies), history of diabetic ketoacidosis (DKA) at the time of initial diagnosis, or low C‐peptide levels (≤0.6 ng/mL in the fasting state or ≤1.8 ng/mL postprandially). In addition, all patients were receiving insulin therapy.

**Figure 1:**
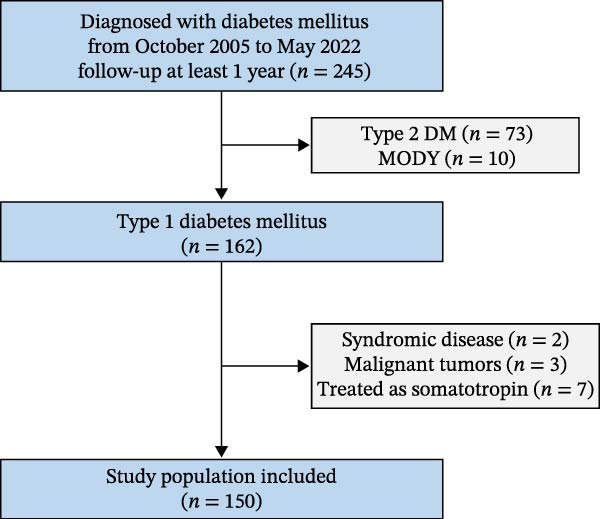
Study selection and baseline population. DM, diabetes mellitus; MODY, maturity‐onset diabetes of the young.

Seventy‐three patients diagnosed with type 2 diabetes and 10 patients with maturity‐onset diabetes of the young (MODY) were excluded. All MODY cases were genetically confirmed using a next‐generation sequencing (NGS) panel targeting MODY–related genes. Among T1DM patients, two patients with syndromic conditions—Wolfram syndrome and Down syndrome—were excluded from the analysis, as short stature was a common feature of these disorders and may result in growth patterns that differ from those of the general population. Three patients with malignant tumors that could affect growth were excluded. Seven patients treated with somatotropins were also excluded (Figure 1).

The HbA1c levels were checked at 3‐month intervals, with values from the time of diagnosis up to 3 months excluded. The adjusted HbA1c–standard deviation (SD), which reflected visit‐to‐visit variability in HbA1c, was used to evaluate long‐term glycemic management variability. To account for the impact of the number of visits (*n*) on the SD of HbA1c, we adjusted the HbA1c‐SD by dividing it by (*n*/[*n* − 1])^0.5^ [19]. We collected the annual height data from the medical records, starting from the onset of diabetes to 5 years postdiagnosis and calculated the H‐SDS according to the growth charts of Korean children in 2017 [20]. Mid‐parental and final heights were collected if available. Clinical data for follow‐up statistical analyses were obtained during a follow‐up period of at least three to 6 months. CGM data were collected using the Dexcom G6 and FreeStyle Libre 1 devices, which were the most recent models available in Korea during the study period.

### 2.2. Measurements

The height was measured using a Harpenden stadiometer (Holtain, Crymych, Dyfed, United Kingdom). The HbA1c was measured at the outpatient clinic using the third‐generation Roche Diagnostics immunoturbidimetric inhibition method using Cobas c 513 (Roche Diagnostics, Mannheim, Germany).

Annual screening for diabetic complications was performed in patients with diabetes for at least 5 years. Nephropathy was confirmed when albumin excretion in the 24‐h urine collection was >30 mg in repeated tests. Peripheral neuropathy was confirmed by a neurologist using autonomic nerve function tests, and sensory and motor nerve conduction velocity tests, which were performed on the median, ulnar, tibial, sural, and common peroneal nerves using standard methods [1]. Diabetic retinopathy was diagnosed by an ophthalmologist using dilated fundus examination.

### 2.3. Statistical Methods

Continuous variables are presented as mean ± SD for normally distributed data and as median with interquartile range (IQR) for nonnormally distributed data.

Longitudinal changes in growth parameters, including H‐SDS, weight‐SDS (W‐SDS), and body mass index‐SDS (BMI‐SDS), were analyzed using linear mixed‐effects models (LMM). Time was defined as the duration from diagnosis to each repeated measurement and was treated as a continuous variable to estimate the rate of change over the 5‐year period following diagnosis. Fixed effects included time, glycemic control group (well‐controlled vs. poorly controlled), CGM use, and relevant interaction terms. Random intercepts were specified for each participant to account for within‐subject correlation.

Multivariable analyses evaluating factors associated with longitudinal changes in H‐SDS were conducted using the same mixed‐effects framework. Model parameters were estimated using restricted maximum likelihood (REML), and the random‐effects structure was selected based on the Akaike information criterion (AIC). Model coefficients were interpreted as estimated annual rates of change, with standard errors (SEs) reported accordingly. Model assumptions were assessed using standard diagnostic procedures, including residual *Q*–*Q* plots, which did not indicate substantial deviations from normality. Missing follow‐up measurements were handled using an observed‐case approach, with all available observations included in the analyses without imputation.

Effect estimates are presented with 95% confidence intervals (CI) as measures of precision. All statistical analyses were performed using R version 4.4.1 and SAS version 9.4 (SAS Institute Inc., Cary, NC). All *p*‐values were two‐sided, and statistical significance was defined as *p*  < 0.05.

### 2.4. Ethical Approval

This study was approved by the Institutional Review Board of the Yonsei University College of Medicine (Approval number: 4‐2022‐0821) and was conducted according to the tenets of the Declaration of Helsinki. The requirement for informed consent was waived because of the retrospective study design and the minimal risk to the subjects.

## 3. Results

### 3.1. Baseline Characteristics

A total of 150 participants were included (45.3% male). Baseline characteristics were summarized in Table 1. There were no significant sex differences in age at diagnosis or follow‐up duration. Females showed significantly higher mean HbA1c (8.2% ± 1.5% vs. 7.7% ± 1.7%, *p* = 0.03) and greater HbA1c variability (0.9 ± 0.5 vs. 0.7 ± 0.5, *p*  < 0.01), indicating poorer glycemic control. Pubertal onset at diagnosis was more common in females (47.0% vs. 30.9%, *p* = 0.03). CGM use (27.2%) and complication rates (50.9%) did not differ significantly by sex. Seventy‐eight patients (52.0%) attained final adult height.

**Table 1:** Baseline characteristics of the study population.

Variables	Total (*N* = 150)	Male (*N* = 68, 45.3%)	Female (*N* = 82, 54.7%)	*p*‐Value
Age at diagnosis (years)	7.8 ± 3.6	7.5 ± 3.5	7.9 ± 3.6	0.48
Follow‐up period (years)	7.1 ± 4.6	6.4 ± 4.3	7.7 ± 4.8	0.07
Puberty (%)	60 (40.0%)	21 (30.9%)	39 (47.6%)	**0.03**
Mean HbA1c (%)^a^	8.0 ± 1.6	7.7 ± 1.7	8.2 ± 1.5	**<0.01**
Adjusted HbA1c‐SD (HbA1c variability; %)	0.8 ± 0.5	0.7 ± 0.4	0.9 ± 0.5	**<0.01**
Mid‐parental height (cm)	167.0 ± 7.6	173.8 ± 3.9	161.3 ± 4.7	**<0.01**
Final adult height (cm)^b^	165.9 ± 8.1	173.1 ± 6.8	162.3 ± 6.2	**<0.01**
Height at diagnosis (cm)	129.2 ± 24.8	128.8 ± 2.56	129.5 ± 24.3	0.79
Height after 5‐year diagnosis (cm)^c^	150.1 ± 18.1	153.4 ± 18.2	147.3 ± 17.7	0.08
Height at diagnosis (H‐SDS)	0.38 ± 1.11	0.53 ± 1.05	0.24 ± 1.14	0.11
Height after 5‐year diagnosis (H‐SDS)^c^	0.22 ± 1.25	0.19 ± 1.07	0.25 ± 1.40	0.83
Weight at diagnosis (W‐SDS)	−0.14 ± 1.05	0.04 ± 1.01	−0.30 ± 1.06	0.06
Body mass index at diagnosis (BMI‐SDS)	−0.51 ± 1.12	−0.30 ± 1.08	−0.69 ± 1.12	**0.046**
Continuous glucose monitoring (*N*, %)	41 (27.3%)	19 (27.9%)	22 (26.8%)	0.86
Complications	77 (51.3%)	37 (54.4%)	40 (48.8%)	0.51
Nephropathy	16 (10.7%)	5 (7.4%)	11 (13.4%)	0.3
Neuropathy	65 (43.3%)	34 (50.0%)	31 (37.8%)	0.14
Retinopathy	31 (20.7%)	16 (23.5%)	15 (18.3%)	0.43

*Note:* The values highlighted in bold indicate statistically significant *p*‐values. Data are shown as mean ± SD. HbA1c, glycosylated hemoglobin.

Abbreviations: BMI‐SDS, body mass index‐standard deviation score; H‐SDS, height‐standard deviation score; SD, standard deviation; W‐SDS, weight‐standard deviation score.

^a^Mean HbA1c ≥7.0% in 113 participants (75.3%), of whom 43 (38.1%) were male.

^b^The population included in this analysis consisted of 78 (52.0%) individuals.

^c^The population included in this analysis consisted of 86 (57.3%) individuals, with 40 (46.5%) males.

### 3.2. Linear Growth After Diagnosis of T1DM

**Figure 2:**
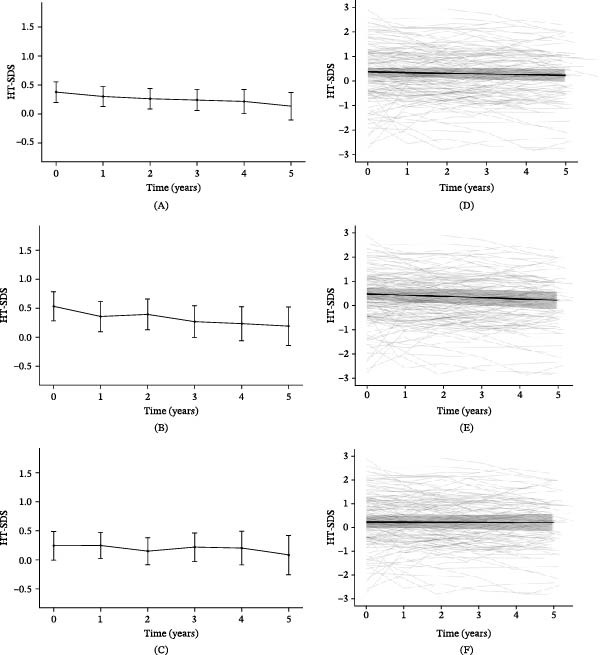
Mean height SDS and individual trajectories from diagnosis to 5 years after diagnosis. Mean height SDS overtime in the (A) total cohort, (B) males, and (C) females. Individual trajectories of height SDS for the (D) total cohort, (E) males, and (F) females, based on a linear mixed‐effects model with random intercepts and slopes. Shade represents the 95% confidence intervals. SDS, standard deviation score.

Although the absolute values of height and weight increased overtime, H‐SDS decreased (Figure 2A), while W‐SDS showed an increasing trend. At the time of diagnosis, the mean BMI was 16.53 ± 2.90, indicating an underweight status. It increased to 17.92 ± 3.11 at 1 year, 18.40 ± 3.04 at 2 years, 18.72 ± 2.91 at 3 years, and 19.21 ± 3.10 at 4 years after diagnosis, entering the normal range after the second year. In terms of BMI‐SDS, the mean value at diagnosis was −0.54 ± 1.14, corresponding to the lower end of the normal range. By 1 year after diagnosis, it had increased to 0.04 ± 0.96 and continued to show a gradual upward trend thereafter. At the time of T1DM diagnosis, 12 patients (8.6%) were classified as overweight or obese. Of these, 10 patients remained in the same category throughout the follow‐up period. By 5 years postdiagnosis, the number of patients classified as overweight or obese had increased to 19 (22.9%).

After diagnosis, H‐SDS showed a slight, but statistically significant decrease overtime, with an estimated slope (*β*) of −0.029 (SE = 0.009, 95% CI: −0.045 to −0.012, *p*  < 0.01; Figure 2D). In contrast, both W‐SDS and BMI‐SDS increased slightly overtime (W‐SDS: *β* = 0.073, SE = 0.011, 95% CI: 0.05 to 0.094, *p*  < 0.01; BMI‐SDS: *β* = 0.120, SE = 0.014, 95% CI: 0.093–0.146, *p*  < 0.01; Supporting Information 1).

### 3.3. Linear Growth According to Sex After Diagnosis of T1DM

We analyzed the H‐SDS after the diagnosis of T1DM according to sex. The H‐SDS in males was 0.53 ± 1.05 at the time of diagnosis, 0.40 ± 1.09 at 1 year, and 0.19 ± 1.07 at 5 years after diagnosis, indicating a gradual decline overtime (Figure 2B). As analyzed using a LMM, H‐SDS in males significantly decreased overtime (*β* = −0.054, SE = 0.013, 95% CI: −0.079 to −0.029, *p*  < 0.01; Figure 2E), while BMI‐SDS showed a significant increase (*β* = 0.059, SE = 0.019, 95% CI: 0.023 to 0.096, *p*  < 0.01). No significant change was observed in W‐SDS overtime.

In females, the H‐SDS at the time of diagnosis was 0.31 ± 1.21 and slightly increased as 0.34 ± 1.12 at 1 year after diagnosis, but decreased trend through 5 years after diagnosis as 0.25 ± 1.40 (Figure 2C). As analyzed using a LMM, H‐SDS in females showed no significant change overtime after diagnosis (Figure 2F). In contrast, both W‐SDS and BMI‐SDS increased significantly (W‐SDS: *β* = 0.121, SE = 0.015, 95% CI: 0.093 to 0.149, *p*  < 0.01; BMI SDS: *β* = 0.174, SE = 0.019, 95% CI: 0.136 to 0.211, *p*  < 0.01). The BMI‐SDS at diagnosis was lower in females (−0.74 ± 1.16) than in males (−0.30 ± 1.08); however, the trajectory following diagnosis was similar between sexes (Supporting Information 1).

### 3.4. Linear Growth According to the HbA1c Levels After Diagnosis of T1DM

**Figure 3:**
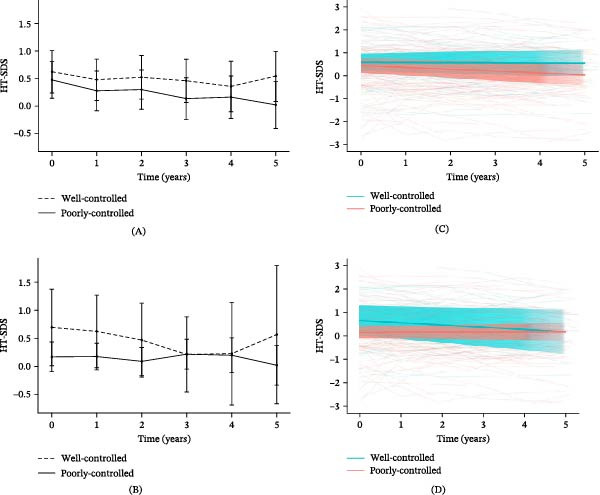
Mean height SDS and individual trajectories from diagnosis to 5 years after diagnosis according to HbA1c levels. Mean height SDS overtime in the (A) males and (B) females. Individual trajectories of height SDS for the (C) males and (D) females, based on a linear mixed‐effects model with random intercepts and slopes. Among males, the numbers in the well‐controlled group were 27, 26, 26, 24, 18, and 13 at baseline and years 1–5, respectively, and 41, 36, 35, 34, 30, and 27 in the poorly‐controlled group. Among females, the corresponding numbers were 11, 11, 11, 10, 7, and 5 in the well‐controlled group and 71, 60, 62, 58, 50, and 41 in the poorly‐controlled group. All available observations were included in the linear mixed‐effects model. Shade represents the 95% confidence intervals. HbA1c G1 indicates the well‐controlled group (mean HbA1c <7.0%, *n* = 37), and HbA1c G2 indicates the poorly controlled group (mean HbA1c ≥7.0%, *n* = 114). HbA1c, glycosylated hemoglobin. SDS, standard deviation score.

Of the 150 participants, 37 (24.7%) were classified as well‐controlled (mean HbA1c <7.0%) and 113 (75.3%) as poorly‐controlled (mean HbA1c ≥7.0%) over the follow‐up period. (Figure 3). In males (Figure 3A), the H‐SDS of the well‐controlled group demonstrated higher H‐SDS trends overall, and the H‐SDS at 5 years postdiagnosis compared to baseline was greater (∆H‐SDS per 5 year, −0.05 ± 0.57 vs. −0.14 ± 0.96, *p* = 0.69). Mid‐parental height (MPH) did not differ significantly between well‐controlled and poorly controlled groups in either sex, with comparable values observed in both males (173.3 ± 3.7 cm vs. 173.6 ± 4.0 cm) and females (160.1 ± 3.4 cm vs. 161.5 ± 4.9 cm).

In the poorly controlled group of males, H‐SDS showed a significantly greater decrease compared to the well‐controlled group (well‐controlled: *β* = −0.007, SE = 0.020, 95% CI: −0.047 to −0.033; poorly‐controlled: *β* = −0.081, SE = 0.016, 95% CI: −0.112 to −0.050; *p*  < 0.01; Figure 3C). W‐SDS and BMI‐SDS showed slightly greater increases in the well‐controlled group (W‐SDS: well‐controlled, *β* = 0.063, SE = 0.028, 95% CI: 0.014–0.114; poorly‐controlled, *β* = –0.006, SE = 0.021, 95% CI: −0.051 to 0.025; *p* = 0.02; BMI‐SDS: well‐controlled, *β* = 0.110, SE = 0.030, 95% CI: 0.049 to 0.168; poorly‐controlled, *β* = 0.030, SE = 0.023, 95% CI: −0.016 to 0.076; *p* = 0.04; Supporting Information 2).

Among females, the H‐SDS slightly decreased at 1‐year postdiagnosis. In the poorly‐controlled group, the H‐SDS decreased at 3 years and then recovered at 4 to 5 years postdiagnosis, with no significant differences observed. Nonetheless, the well‐controlled group maintained higher H‐SDS than did the poorly controlled group throughout the study period (Figure 3B). There were no significant differences between the two groups in H‐SDS (Figure 3D), W‐SDS, or BMI‐SDS based on the LMM (Supporting Information 2).

### 3.5. Linear Growth According to the Use of CGM After Diagnosis of T1DM

**Figure 4:**
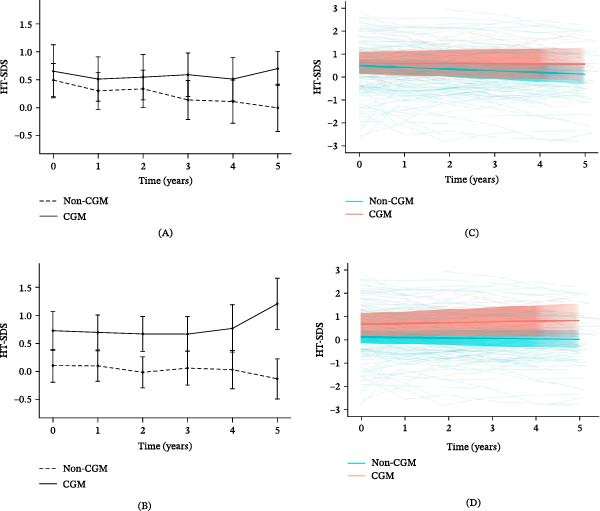
Mean height SDS and individual trajectories from diagnosis to 5 years after diagnosis according to CGM use. Mean height SDS overtime in the (A) males and (B) females. Individual trajectories of height SDS for the (C) males and (D) females, based on a linear mixed‐effects model with random intercepts and slopes. Among males, the numbers in the non‐CGM group were 51, 45, 44, 41, 33, and 29 at baseline and years 1–5, respectively, and 17, 17, 17, 17, 15, and 11 in the CGM group. Among females, the corresponding numbers were 64, 54, 56, 51, 44, and 39 in the non‐CGM group and 18, 17, 17, 17, 13, and 7 in the CGM group. All available observations were included in the linear mixed‐effects model. Shade represents the 95% confidence intervals. CGM, continuous glucose monitoring; SDS, standard deviation score.

We analyzed the H‐SDS after the diagnosis of T1DM in relation to the use of CGM (Figure 4). The mean HbA1c level was significantly lower in the CGM group compared to the non‐CGM group (7.18 ± 1.02% vs. 8.20 ± 1.69%, *p*  < 0.01). Additionally, HbA1c variability was lower in the CGM group (0.50 ± 0.37) than in the non‐CGM group (0.86 ± 0.50, *p*  < 0.01). MPH did not differ significantly between CGM and non‐CGM groups in either sex, with values of 174.8 ± 3.2 cm vs. 173.3 ± 4.1 cm in males and 161.0 ± 4.1 cm vs. 161.4 ± 5.0 cm in females.

The H‐SDS was consistently higher in the CGM group than in the non‐CGM group. For males in the CGM group, the H‐SDS remained positive based on the age‐ and sex‐specific reference [20] throughout the 5‐year follow‐up period after the diagnosis. In contrast, in the non‐CGM group, the H‐SDS decreased significantly over the 5‐year follow‐up period after the diagnosis (Figure 4A).

In males, at the 5‐year follow‐up after diagnosis, the CGM group showed significantly higher H‐SDS overtime compared to the non‐CGM group (CGM: *β* = −0.012, SE = 0.023, 95% CI: −0.057 to 0.034; non‐CGM: *β* = −0.072, SE = 0.015, 95% CI: −0.101 to −0.042; *p* = 0.03; Figure 4C). In females using CGM, H‐SDS showed a steady increase compared to non‐CGM group (Figure 4B); however, there was no significant difference overtime between the two groups (CGM: *β* = 0.035, SE = 0.026, 95% CI: −0.014 to 0.084; non‐CGM: *β* = −0.018, SE = 0.013, 95% CI: −0.043 to 0.008; *p* = 0.16; Figure 4D). W‐SDS and BMI‐SDS showed no significant differences between the two groups (Supporting Information 3).

### 3.6. Multivariable Factors Affecting the Changes in H‐SDS Between the Time of Diagnosis and the 5‐Year Mark After the Diagnosis

We used LMM to evaluate factors associated with changes in H‐SDS from diagnosis to 5 years after diagnosis. In univariable analyses, older age at diagnosis, female sex, higher mean HbA1c, pubertal status at diagnosis, and use of CGM were significantly associated with changes in H‐SDS (Table 2). In the multivariable LMM, older age at diagnosis remained independently associated with a smaller gain in H‐SDS (*β* = −0.009, 95% CI −0.017 to −0.002; *p* = 0.02). Female sex was associated with a greater increase in H‐SDS compared with males (*β* = 0.067, 95% CI: 0.033 to 0.100; *p*  < 0.01). Higher mean HbA1c across follow‐up was consistently and independently inversely associated with growth (*β* = −0.026, 95% CI: −0.038 to −0.015; *p*  < 0.01).

**Table 2:** Linear mixed model analysis of the differences in H‐SDS.

Effect of the differences in H‐SDS (0–5 years)	Univariable LMM		Multivariable LMM	
	Beta (95% CI)	*p*‐Value	Beta (95% CI)	*p*‐Value
Age at diagnosis	−0.014 (−0.019 to –0.009)	**<0.01**	−0.009 (−0.017 to −0.002)	**0.02**
Sex	0.047 (0.014 to 0.080)	**<0.01**	0.067 (0.033 to 0.100)	**<0.01**
Mid‐parental height	−0.001 (−0.003 to 0.001)	0.40	—	—
Mean HbA1c^a^	−0.024 (−0.035 to −0.013)	**<** **0.01**	−0.026 (−0.038 to −0.015)	**<0.01**
Puberty	−0.083 (−0.018 to −0.047)	**<0.01**	−0.034 (−0.088 to 0.020)	0.22
Continuous glucose monitoring^a^	0.050 (0.012 to 0.089)	**0.01**	0.019 (−0.021 to 0.059)	0.35
Complication	0.023 (−0.010 to 0.057)	0.17	—	—

*Note:* The values highlighted in bold indicate statistically significant *p*‐values. HbA1c, glycosylated hemoglobin.

Abbreviations: CI, confidence interval; H‐SDS, height‐standard deviation score; LMM, linear mixed model.

^a^No significant interaction was observed between CGM use and mean HbA1c (CGM × HbA1c interaction *p* = 0.72).

Although pubertal status at diagnosis showed a significant negative association with H‐SDS change in the univariable model, this association was no longer significant after multivariable adjustment. Similarly, CGM use was associated with greater H‐SDS change in univariable analysis, but did not remain significant in the adjusted model (*β* = 0.019, 95% CI: −0.021 to 0.059; *p* = 0.35). To further examine the influence of glycemic control and CGM use, we performed stratified multivariable analyses in CGM and non‐CGM groups. In both groups, age at diagnosis and puberty remained a significant predictor of growth. Mean HbA1c was also negatively associated with H‐SDS change in the both groups; however, the association did not reach statistical significance after adjustment, suggesting that CGM use itself was not an independent modifier of the growth response (CGM × HbA1c interaction *p* = 0.72, Supporting Information 4).

## 4. Discussion

This study provides important insights into the growth patterns and factors associated with linear growth in patients with T1DM. Longitudinal analysis of the H‐SDS, stratified by sex, glycemic control, and CGM use, revealed that good glycemic control was associated with more favorable H‐SDS trajectories after diagnosis.

Our findings indicate that at the time of diagnosis, males and females had positive baseline H‐SDS values. Over the first year following diagnosis, both males and females showed a decrease in H‐SDS, although males exhibited more significant decreases in H‐SDS, which continued to decline over subsequent years. This result is consistent with previous studies [12] from Germany and Austria, which observed that children with T1DM had an average H‐SDS of 0.22 ± 1.00 at diagnosis, reflecting positive standardized height scores relative to the reference. In the Diabetes Autoimmunity Study in the Young cohort, a higher height velocity was positively correlated with the onset of islet autoimmunity (hazard ratio [HR]: 1.63, 95% CI: 1.31 to 2.05) and progression to T1DM (HR: 3.34, 95% CI: 1.73 to 6.42), which resulted in an H‐SDS that was above the average at the time of diagnosis [21].

Impaired growth velocity, evidenced by a decline in H‐SDS from the disease onset to the 5‐year height change, has been consistently observed in other cohorts of patients with T1DM and is consistent with the findings of our study [2, 9, 22, 23]. In our study, decreased growth velocity was more pronounced in males. Previous studies [23–25] have suggested that sex hormones, including estrogen, may contribute to sex‐specific differences in growth patterns, but we were unable to assess these hormonal mechanisms because such biomarkers were not collected in our cohort. Although H‐SDS decreased overtime and W‐SDS increased relatively, resulting in an overall increase in BMI. In our cohort, the prevalence of elevated weight status rose from 8.6% at diagnosis to 22.9% after 5 years, alongside a mean BMI‐SDS increase from –0.54 to 0.04. Most individuals with excess weight at baseline remained in the same category during follow‐up, suggesting that early deviations in weight trajectory tend to persist. This pattern is consistent with trends seen in other pediatric populations with T1DM [26, 27]. The progressive rise in weight highlights the importance of incorporating structured nutritional support and promoting physical movement as part of long‐term metabolic care [28]. This may reflect the typical clinical presentation of T1DM at diagnosis, where significant weight loss is often observed. Thereafter, BMI increased after diagnosis, which may be attributed to the anabolic effects of insulin therapy [26].

The analysis of the H‐SDS trends based on the HbA1c levels demonstrated significant differences between well‐controlled (HbA1c <7.0%) and poorly controlled (HbA1c ≥7.0%) groups, especially in males. Well‐controlled patients maintained higher H‐SDS over the 5‐year follow‐up period. This finding aligns with that of many previous studies, indicating that tight glycemic control is crucial for maintaining normal growth in children with T1DM [23, 29–33] with a trend towards a greater decline in males, similar to other studies [30, 34]. Also, the well‐controlled group showed greater weight gain overtime and BMI also increased significantly, remaining within the normal range. This suggests that appropriate insulin treatment led to effective glycemic control, which in turn supported healthy weight gain consistent with expected growth patterns.

When examining the impact of CGM use, our findings revealed that both males and females using CGM had higher H‐SDS, with males exhibiting greater changes in H‐SDS over the 5‐year follow‐up period compared to those not using CGM. Given that the HbA1c levels were lower in the CGM group, it can be inferred that improved glycemic control achieved through CGM use has a positive influence on the H‐SDS. Even in the group of CGM users, the H‐SDS increased after diagnosis. Many studies [35] have revealed that the use of CGM has shown better glycemic control, especially lower HbA1c levels, in children (*p*  < 0.01). By utilizing multiple daily injections or continuous subcutaneous insulin infusion to effectively manage HbA1c levels, the GH/IGF‐1 axis can be maintained within the normal range [8]. In this context, our study underscores that CGM use was associated with more favorable H‐SDS trajectories, primarily through improved glycemic control. These findings suggest that CGM use may be associated with more favorable growth trajectories, primarily through its contribution to improved metabolic control.

By analyzing multivariable LMM, our findings suggest that age at diagnosis, sex, and long‐term metabolic control, rather than pubertal status were the key factors associated with the 5‐year change in H‐SDS. In contrast to prior study [12] reporting greater growth impairment in children diagnosed at younger ages, particularly when near‐adult or final height is considered, we observed a smaller gain in H‐SDS with increasing age at diagnosis. This discrepancy is likely attributable to differences in follow‐up duration, as previous reports [12] primarily reflect the cumulative impact of long‐standing diabetes on ultimate height, whereas our analysis was limited to growth changes within the first 5 years after diagnosis. During this early postdiagnostic period, younger children may retain greater potential for catch‐up growth following metabolic stabilization, while those diagnosed at older ages may have a more constrained window for growth recovery. These findings suggest that the effect of age at diagnosis on growth trajectories is time dependent, differing between early postdiagnostic growth and long‐term height outcomes [2]. Several longitudinal studies [2, 36] have reported that children with T1DM frequently present higher mean HbA1c over the 5‐year period was also independently associated with greater decline in H‐SDS, underscoring the contribution of chronic metabolic control to longitudinal growth. This aligns with previous evidence linking persistent hyperglycemia to impaired growth [23, 32]. Although we did not observe a significant difference in final height between HbA1c subgroups, likely due to incomplete final height data, the observed association between mean HbA1c and H‐SDS change is consistent with established literature. Sex‐specific differences were also observed, with females demonstrating a greater increase in H‐SDS compared with males after multivariable adjustment, consistent with prior reports of differential growth responses between sexes in T1DM [24, 32]. Although CGM use was associated with greater changes in H‐SDS in univariable analyses, this association was attenuated after multivariable adjustment, and CGM did not modify the relationship between HbA1c and growth. These findings indicate that CGM use is unlikely to exert an independent or differential effect on linear growth. Instead, its clinical relevance may primarily relate to supporting glycemic management, with cumulative metabolic control remaining the dominant determinant of long‐term growth trajectories.

The overall complication rate was relatively high at 50.9%. This may be attributed to the long follow‐up duration, with 29 patients (19.3%) having a disease duration of over 10 years. In addition, only 25.3% of patients were classified as well‐controlled (HbA1c ≤7%), suggesting that suboptimal glycemic control may have contributed to the higher complication rate. Furthermore, the complication assessment included abnormal findings on autonomic nervous conduction tests, even in the absence of clinical symptoms. As a result, the true prevalence may have been slightly overestimated.

This study has several limitations. First, as this was a single‐center study, the number of patients included was relatively small, and the proportion of patients using CGM was less than half, given its widespread adoption only recently. Second, detailed information on CGM use, such as wear‐time adherence thresholds and replacement schedules, was unavailable due to the retrospective nature of the study. This limitation may have hindered a more causative interpretation of the observed outcomes. However, our study has novelty and strength in its relatively long follow‐up period, which examined linear growth in patients with T1DM. Although some associations did not reach statistical significance after multivariable adjustment, the overall increase in the H‐SDS among CGM users with T1DM suggests a potential positive impact on linear growth. With the accumulation of more long‐term data and larger sample sizes, these findings can be substantiated in future studies.

## 5. Conclusion

Good glycemic control positively influenced the H‐SDS after the diagnosis of T1DM. The use of CGM may support better linear growth even at lower HbA1c levels in children with T1DM.

## Author Contributions

Sujin Kim made substantial contributions to the conception and design, interpretation of data, and drafting of the manuscript. Junghwan Suh and Kyungchul Song contributed to the conception and execution of the study. Junghwan Suh and Hyun Wook Chae made contributions to the conception and design of the study. Seo Jung Kim and Hyun Wook Chae contributed to the preparation of data and figures. Kyungchul Song, Youngha Choi, and Hyun Wook Chae contributed to the analysis and interpretation of data. Junghwan Suh contributed to the critical revision of important intellectual content.

## Funding

No funding was received for this manuscript.

## Disclosure

The views expressed are those of the authors and not necessarily those of the funders.

## Conflicts of Interest

The authors declare no conflicts of interest.

## Supporting Information

Additional supporting information can be found online in the Supporting Information section.

## Supporting information


**Supporting Information 1** Weight SDS and BMI SDS from diagnosis to after 5 years.

**Supporting Information 2** Weight SDS and BMI SDS from diagnosis to after 5 years according to the HbA1c levels.

**Supporting Information 3** Weight SDS and BMI SDS from diagnosis to after 5 years according to use of the CGM.

**Supporting Information 4** Linear mixed model of the differences in H‐SDS according to use of CGM.

## Data Availability

The data that support the findings of this study are available upon request from the corresponding author. The data are not publicly available due to privacy or ethical restrictions.
