# Supplementary material for: Long‐Term Growth Outcomes of Children With Type 1 Diabetes According to Glycemic Control and Use of Continuous Glucose Monitoring: A Retrospective Cohort Study
Source: Pediatr Diabetes. 2026 Mar 2;2026:9111583. doi: 10.1155/pedi/9111583 (PMC12952282; doi:10.1155/pedi/9111583)
Supplement: Supplementary file 2 — Supporting Information 2 Weight SDS and BMI SDS from diagnosis to after 5 years according to the HbA1c levels. [file PEDI-2026-9111583-s002.docx]

**Supporting information**

1. Weight SDS of (a) males, (b) females and BMI SDS (c) males, (d) females from diagnosis to after 5 years according to the HbA1c levels

| (a) | (b) |
| --- | --- |
| 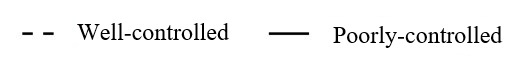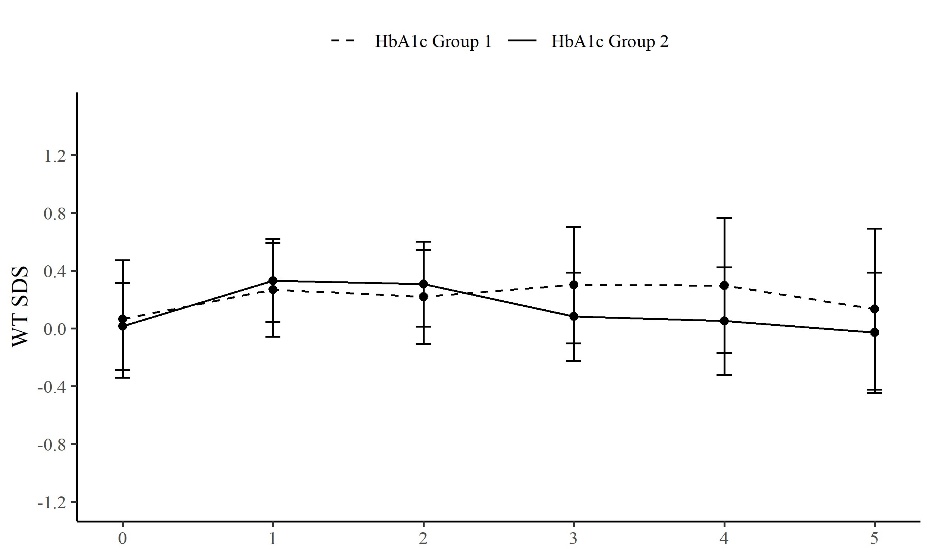 | 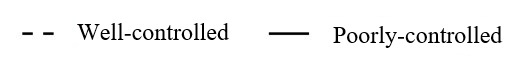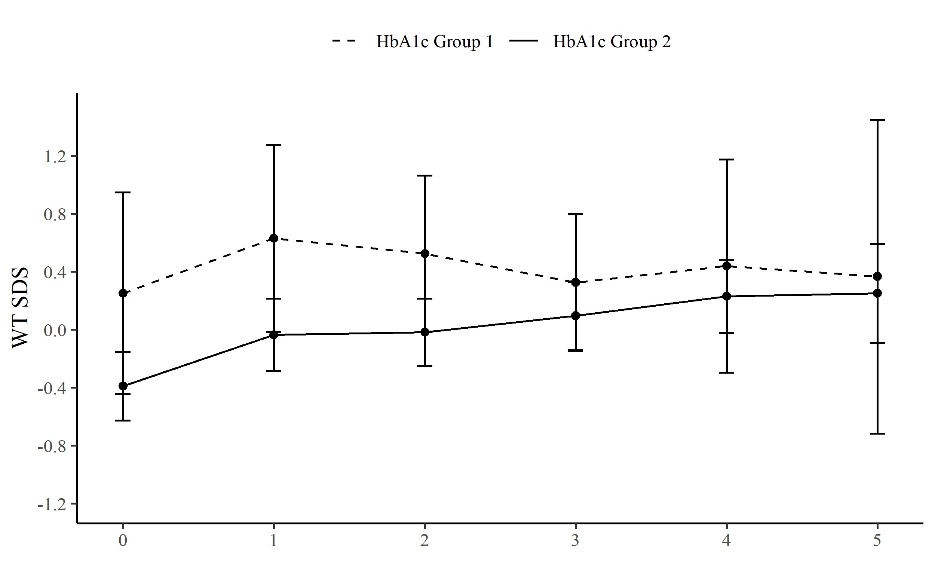 |
| (c) | (d) |
| 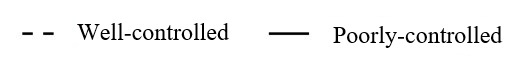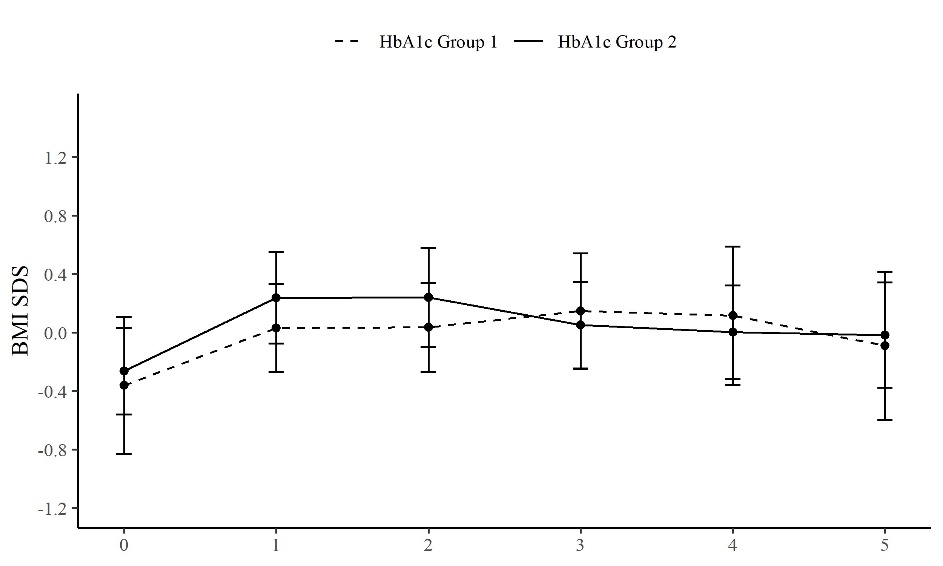 | 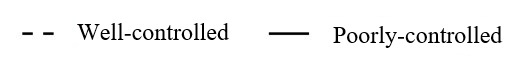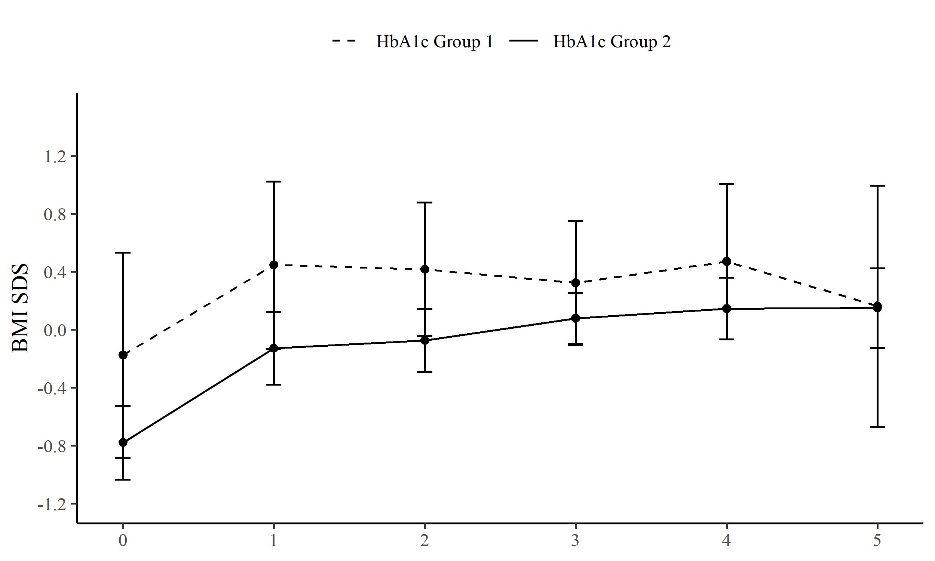 |

Error bars represent the 95% confidence intervals.

SDS, standard deviation score; HbA1c, glycosylated hemoglobin; WT, weight; BMI, body mass index
