# Supplementary material for: Long‐Term Growth Outcomes of Children With Type 1 Diabetes According to Glycemic Control and Use of Continuous Glucose Monitoring: A Retrospective Cohort Study
Source: Pediatr Diabetes. 2026 Mar 2;2026:9111583. doi: 10.1155/pedi/9111583 (PMC12952282; doi:10.1155/pedi/9111583)
Supplement: Supplementary file 4 — Supporting Information 4 Linear mixed model of the differences in H‐SDS according to use of CGM. [file PEDI-2026-9111583-s004.docx]

**Supporting information**

1. Linear mixed model of the differences in H-SDS according to use of CGM

|  | CGM | | | | Non-CGM | | | |
| --- | --- | --- | --- | --- | --- | --- | --- | --- |
|  | Univariable LMM | | Multivariable LMM | | Univariable LR | | Multivariable LMM | |
|  | Beta (95% CI) | p-value | Beta (95% CI) | p-value | Beta (95% CI) | p-value | Beta (95% CI) | p-value |
| Age at diagnosis | –0.021 (–0.030 to –0.011) | <0.01 | –0.020 (–0.033 to –0.007) | <0.01 | –0.011 (–0.017 to –0.006) | <0.01 | –0.003(–0.012 to 0.006) | 0.52 |
| Gender | 0.046 (–0.016 to 0.108) | 0.15 |  |  | 0.054 (0.015 to 0.093) | <0.01 | 0.077 (0.037 to 0.116) | <0.01 |
| MPH | –0.001 (–0.005 to 0.003) | 0.55 |  |  | –0.001 (–0.004 to 0.002) | 0.34 |  |  |
| HbA1c (Mean) | –0.028 (–0.063 to 0.007) | 0.12 |  |  | –0.021 (–0.034 to 0.008) | <0.01 | –0.028 (–0.040 to –0.015) | <0.01 |
| Puberty | –0.103 (–0.173 to –0.033) | <0.01 | –0.041 (–0.132 to 0.049) | 0.37 | –0.073(–0.115 to –0.032) | <0.001 | –0.072(–0.138 to –0.007) | <0.01 |
| Complication | –0.091 (–0.153 to –0.029) | <0.01 | –0.123 (–0.183 to –0.063) | <0.01 | 0.053 (–0.014 to 0.093) | <0.01 | 0.066 (0.027 to 0.104) | <0.01 |

The values highlighted in bold indicate statistically significant p-values.

H-SDS, height-standard deviation score; CGM, continuous glucose monitoring; LMM, linear mixed model; CI, confidential interval; adj, adjusted

MPH, Mid-parental height; HbA1c, glycosylated hemoglobin
